# Attention rhythmically samples multi-feature objects in working memory

**DOI:** 10.1038/s41598-022-18819-z

**Published:** 2022-08-29

**Authors:** Samson Chota, Carlo Leto, Laura van Zantwijk, Stefan Van der Stigchel

**Affiliations:** Helmholtz Institute, Utrecht University, 3584 CS Utrecht, The Netherlands

**Keywords:** Neuroscience, Psychology

## Abstract

Attention allows us to selectively enhance processing of specific locations or features in our external environment while filtering out irrelevant information. It is currently hypothesized that this is achieved through boosting of relevant sensory signals which biases the competition between neural representations. Recent neurophysiological and behavioral studies revealed that attention is a fundamentally rhythmic process, tightly linked to neural oscillations in frontoparietal networks. Instead of continuously highlighting a single object or location, attention rhythmically alternates between multiple relevant representations at a frequency of 3–8 Hz. However, attention cannot only be directed towards the external world but also towards internal visual working memory (VWM) representations, e.g. when selecting one of several search templates to find corresponding objects in the external world. Two recent studies demonstrate that single-feature objects in VWM are attended in a similar rhythmic fashion as perceived objects. Here we add to the literature by showing that non-spatial retro-cues initiate comparable theta-rhythmic sampling of multi-feature objects in VWM. Our findings add to the converging body of evidence that external and internal visual representations are accessed by similar rhythmic attentional mechanisms and present a potential solution to the binding problem in working memory.

## Introduction

While our subjective visual experience might seem like a continuous stream of incoming information, accumulating evidence suggests that perception as well as attention undergo rhythmic fluctuations. Behavioral rhythms are most often found at frequencies of 3 to 6 Hz and 8 to 12 Hz and have been linked to corresponding neural oscillations in the theta (3 to 8 Hz) and alpha (8 to 12 Hz) bands^1^. When attending two objects in our visual field, reaction times to unpredictable targets are modulated as a function of time at a theta frequency^2–5^, potentially as a result of neural competition^6,7^. These periodic modulations of performance are observed for each location individually but are often found to oscillate in antiphase, congruent with a rhythmic spotlight of attention that samples multiple objects alternatingly and sequentially^8,9^. Not only does this rhythmic sampling seem to be the case for object locations but also for non-spatial features such as color or orientation (i.e., feature-based attention^10,11^).

Attention directed towards objects or features in the external world is assumed to facilitate processing at least partially via boosting of sensory signals^12–18^. A similar mechanism might underly the behavioral benefits observed when directing attention towards internal (memorized) representations in visual working memory (VWM). Predictive cues directed towards one or more items in working memory have been shown to increase recall performance, even if cues are presented several seconds after encoding, commonly described as the retro-cue effect (for a review see Souza and Oberauer^19^). A growing number of studies have revealed a strong overlap in the neurophysiological correlates of attention towards external (currently perceived) stimuli and internal working memory processes^20^. This strong overlap is further supported by findings that strongly implicate early sensory cortices in the maintenance of working memory representations, often referred to as the sensory recruitment hypothesis^21–27^. For instance, attentional selection of items in working memory has been shown to bias activity in sensory areas that process these stimuli^28,29^ and to modulate decodability of item features most strongly in early visual cortices^30^.

To summarize, both external visual stimuli and internal VWM representations are encoded at least partially by overlapping neural populations on which attention needs to act. This raises the intriguing possibility that similar or even identical attentional mechanisms might be engaged for external and internal visual representations. Recent work has begun to extend the rhythmic nature of internal attention directed towards VWM items. Peters et al.^31^ instructed participants to memorize four dots that defined the endpoints and hence the outlines of two objects. A subsequent retro-cue directed and reset attention to one of these objects. At densely sampled (200 ms to 1000 ms) but unpredictable timepoints a target was presented that fell within or outside the memorized outlines of the cued or un-cued object. They observed that reaction times (RT) measured as a function of the distance to the cue were rhythmically fluctuating at a frequency of 6 Hz. Furthermore, the RT time series of responses to the cued versus the un-cued object were modulated in antiphase, indicating that object-based attention periodically sampled the two object locations in VWM. Notably reaction times to targets presented at different-object locations were never faster than those to targets on same-object locations. The authors interpreted this as evidence that attention did not completely periodically shift between objects but alternated between the prioritization of one object and neither object. A second study conducted by Pomper and Ansorge^32^ showed that similar fluctuations in performance occurs between two oriented bars in VWM. Importantly, they presented their stimuli sequentially and at fixation, reducing potential influences of rhythmic spatial attention. The efficiency of probe processing fluctuated at a frequency of 6 Hz and an anti-phasic relationship between the accuracy time series of the first and second stimulus was observed.

The recent emergence of studies finding rhythmic behavioral fluctuations in internal attention, closely resembling those found in external attention, suggest a close relationship between both attentional mechanisms and a central involvement of rhythmic brain activity. A potential mechanistic overlap could have wide-ranging implications for our understanding of how the brain selects internal and external information and how this selection is orchestrated by oscillatory activity. Importantly, however, rhythmic object-based attentional fluctuations in working memory have only been shown for items consisting of single features^31,32^. As object-based attention has been strongly implicated in supporting binding of multiple features both during perception and in working memory^33,34^, we set out to further characterize the behavioral correlates of theta-rhythmic attentional sampling in working memory using items consisting of multiple relevant features in a retro-cue paradigm. We hypothesize a rhythmic modulation of the efficacy of specific memory-probe comparisons at a frequency of 3 to 6 Hz following attentional cues. Furthermore, we hypothesize that behavioral time series corresponding to both memory items will be modulated in antiphase, indicative of an alternating attentional sampling of working memory representations.

### Methodological significance

In a recent publication, Brookshire^35^ critically assesses the widely used statistical permutation tests based on random shuffling of the behavior-SOA pairs (time shuffling). This statistical procedure however does not take into account non-periodic autocorrelational structure that might be present in the behavioral data and therefore leads to a large percentage (> 5%) of type-1 errors. Brookshire^35^ proposes an alternative statistical test based on the estimation of autocorrelation models AR(1). These models capture the autocorrelative structure of the original time series and successfully control for type-1 error rate. To test the reliability of our results we analyzed our data using two distinct techniques consisting of (1) traditional preprocessing and time shuffling and (2) preprocessing steps and statistical methods recommended by Brookshire^35^. This also served to provide insights into whether and how the two techniques affected results. Although resulting in partially different outcomes, both analysis techniques revealed comparable rhythmic fluctuations in behavior in the theta range.

## Materials and methods

### Participants

26 participants (aged 19–28, 19 females) with normal or corrected-to-normal vision participated in the experiment. Informed consent forms were signed before the experiment. After completion, participants were compensated with academic credits or 12 euros. The experiment was carried out in accordance with the protocol approved by the Ethics Committee of the Faculty of Social and Behavioural Sciences of Utrecht University and followed the Code of Ethics of the World Medical Association (Declaration of Helsinki).

### Experimental design

Stimuli were presented at a distance of 58 cm with an LCD display (27-inch, 2560 × 1440 resolution, 120 Hz refresh rate) using the Psychophysics Toolbox running in MATLAB (MathWorks). Stimuli consisted of a central fixation cross (diameter = 0.3°), two circular memory stimuli consisting of randomly oriented gratings (diameter = 10°; spatial frequency = 4 cpdva) both of which had a random color selected from opposite phases of the CIELAB color space (48 sampled colors; L = 59, a = 18, b = − 8), a central color cue (0.93°), a probe (diameter = 10°; spatial frequency = 4 cpdva) whose orientation was shifted slightly relative to the same colored memory stimulus, and a Mondrian mask consisting of a colored pattern (diameter = 10°; code from^36^).

**Figure 1:**
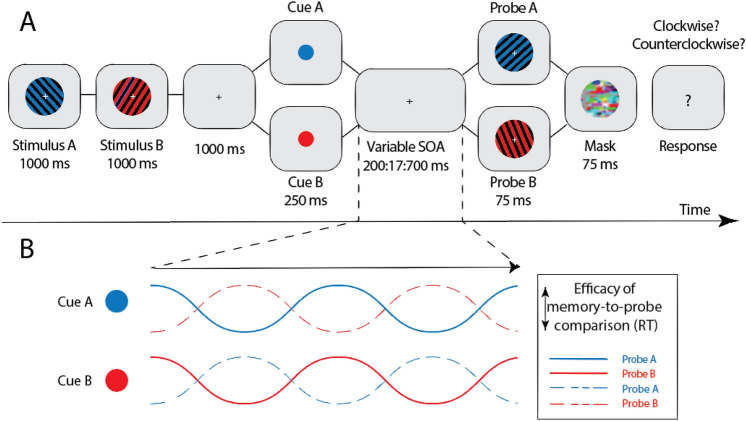
(**A**) Task paradigm. Participants encoded two gratings with different colors and orientations. A subsequent retro-cue, indicative of the probe color (75% validity), was presented to reset attention to one of the items in working memory. We measured behavior at several densely sampled timepoints (between 200 and 700 ms) by presenting the probe and asking participants if it was rotated clockwise or counterclockwise relative to the same-colored item in working memory. (**B**) Expected temporal dynamics of object-based attention in working memory for different cue conditions.

Participants performed a 2AFC working memory task in which they were instructed to compare the orientation of two memory stimuli to the orientation of a probe that matched the color of one of them. Behavior in response to the probe was measured using a dense-sampling approach that allowed us to measure the rhythmic effect of attentional fluctuations on the processing of the probe. Trials began with the presentation of the fixation cross on a blank screen for 1000 ms (Fig. 1). This was followed by the sequential presentation of two memory stimuli for 1000 ms each (Stimulus A and Stimulus B). After a blank delay of 1000 ms, we presented a cue for 250 ms that matched the color of either Stimulus A or Stimulus B and indicated which of the two working memory items will be probed with 75% validity. Based on previous studies we hypothesized that the cue would reset rhythmic attentional sampling while still requiring participants to maintain a representation of the un-cued item^2,4,10,31^. The cue was followed by a blank screen of variable delay (between 200 and 700 ms in steps of 16.7 ms) after which we presented a Probe stimulus for 75 ms that matched either Stimulus A or Stimulus B in color and was oriented slightly clockwise or counterclockwise. After another brief delay of 25 ms, we presented a mask for 75 ms after which participants responded if the orientation of the Probe was tilted clockwise or counterclockwise relative to the same-colored WM stimulus. Participants responded by pressing either the Q (counterclockwise) or the P button (clockwise).

The experiment consisted of a familiarization block (~ 4 min) in which participants performed several trials with binary feedback on every trial. This was followed by a staircase procedure (QUEST, 80 trials, ~ 10 min) that allowed us to tailor the angular difference in orientation between memory item and probe so that performance was on average at 75%^37^. During this procedure participants received binary feedback at the end of every trial. After completion of the staircase procedure the main experiment began (20 blocks of 30 trials each, ~ 75 min). The main experiment was identical to the staircase procedure with the only exception being that feedback was now provided at the end of every block in the form of percent correct instead of at the end of every trial. This was done to minimize training effects. During the main experiment we used a fixed angular difference between sample and probes which was determined during the staircase procedure.

### Data analysis

We only considered trials with reaction times shorter than 2 s. For two participants this led to the exclusion of more than 20% of the trials and they were hence excluded from the analysis. For another participant our RT criteria led to the removal of all trials in a specific SOA, and they were also removed from the analysis. 23 participants were included in the final analysis. Group average reaction times and accuracy were analysed using two sample t-tests. To investigate rhythmic fluctuations in behavior we averaged reaction times within each of 30 succeeding SOAs for each individual participant. This allowed us to compute behavioral time series that reflect dynamic changes in the efficacy of probe comparisons (RT and accuracy) over the maintenance interval. The length of the time window (500 ms) and spacing of SOA’s (sampling frequency 60 Hz) were chosen so that frequencies between 2 and 30 Hz could be analyzed. Only correct trials were considered since they most likely reflect successful working memory maintenance. As we hypothesized that the cue would reset attentional sampling to the cued item, irrespective of its serial position, we calculated the RT time series for validly cued probes and invalidly cued probes separately. The following section will outline subsequent steps in our original analysis pipeline and the novel analysis pipeline respectively.

### Original analysis pipeline (time shuffling)

Individual RT time series were detrended by fitting and removing a 2nd order polynomial and moving averages were calculated using a 66.7 ms window. We then z-scored the RT time series and applied a Hamming window before performing spectral analysis using fast Fourier transformation. The resulting individual power spectra were averaged, and we statistically tested for oscillatory peaks using a non-parametric permutation test. We decided to perform spectral analysis on individual time series (instead of grand-average time series) because individuals’ behavioral rhythms might slightly differ in phase and would hence disappear when averaging all subjects time series in the time domain. We repeated the above-described preprocessing procedure 10.000 times, however in every iteration we randomly shuffled the SOA-RT pairs for every participant. This procedure allowed us to estimate the distribution of power spectra under the null hypothesis that no temporal structure is present in individual RT time series. The real power-spectrum was then compared to the surrogate distribution of power spectra on a frequency-by-frequency basis. Frequencies at which the real power-spectrum exceeded the 95% percentile of the surrogate distribution were considered significant as this equates to an alpha level of 0.05%. We performed a similar analysis on the difference wave between valid and invalid RT time series. Since we were mainly interested in low frequency fluctuations, we only considered frequencies between 1 and 15 Hz.

### Analysis pipeline suggested by Brookshire^35^

Individual time series were smoothed by calculating moving averages using a 66.7 ms window. The individual time series were then detrended linearly before performing spectral analysis using Fast Fourier transform without applying zero-padding or windowing. Individual power spectra were then averaged. We decided to perform spectral analysis on individual time series (instead of grand-average time series) because individuals’ behavioral rhythms might slightly differ in phase and would hence disappear when averaging all subjects time series in the time domain. To test if individual time series fluctuate periodically we fitted an autoregressive model with a single parameter AR(1) using maximum likelihood to individual smoothed and detrended time series. For each participants’ AR(1) model we used Monte-Carlo simulation to generate 5000 time series with the same length, autocorrelation parameter and residual variance as the original time series. We then performed spectral analysis using Fast Fourier transformation on these time series without applying zero-padding or windowing. This procedure allowed us to estimate the distribution of power spectra under the null hypothesis that no rhythmic fluctuation is present in individual time series. The real averaged power-spectrum was then compared to the surrogate distribution of averaged power spectra on a frequency-by-frequency basis. Frequencies at which the real power-spectrum exceeded the 95% percentile of the surrogate distribution were considered significant as this equates to an alpha level of 0.05%. We performed correction for multiple comparisons using false discovery rate. Since we were mainly interested in low frequency fluctuations, we only considered frequencies between 1 and 15 Hz.

For a visual comparison of both analysis pipelines, we refer the reader to Fig. 1 in the Supplementary Materials.

To compare the relative temporal dynamics of attentional fluctuations following valid and invalid cues, oscillatory phases were extracted from the complex components of the Fast Fourier transform of the individual preprocessed time series. To investigate whether individual valid and invalid time series were fluctuating in antiphase we subtracted the phase values and performed Rayleigh’s test of non-uniformity as well as a circular t-test to compare average phase-difference values to 180°.

## Results

**Figure 2:**
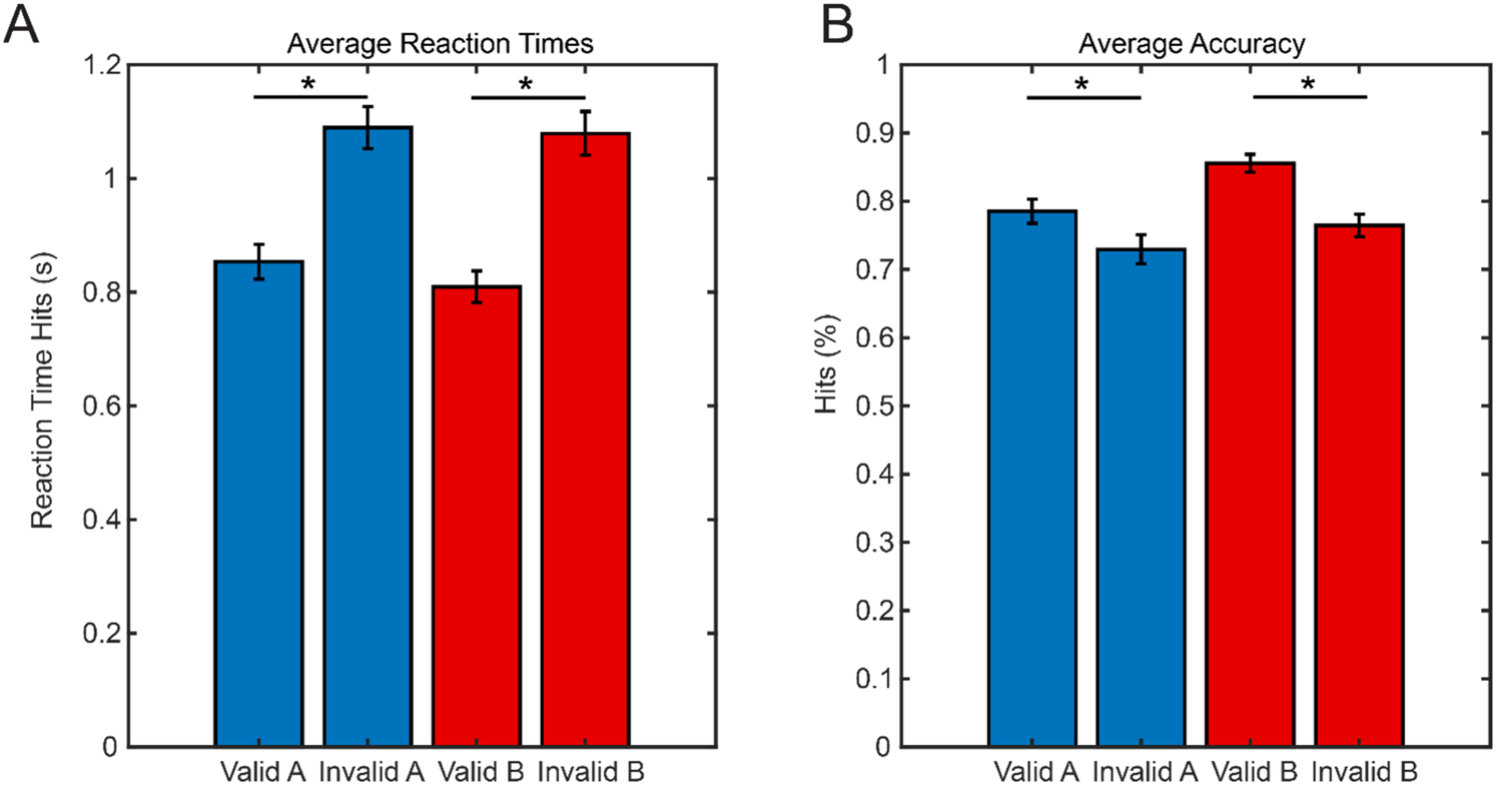
Average reaction times (**A**) and accuracy (**B**) for valid and invalid conditions.

Statistical analysis of the group average RT revealed that participants responded significantly faster to cued (mean = 831 ms) versus un-cued probes (mean = 1084 ms), t (22) = − 12.97, p < 0.001, Fig. 2A). Furthermore, we found that reaction times were shorter when participants responded to probes matching the second stimulus (mean = 840 ms) as compared to the first stimulus (mean = 880 ms, t(22) = 3.85, p < 0.005). Similarly, accuracy was higher for cued probes (mean = 81.1%) than for uncued probes (mean = 73.2%, t(22) = 8.76, p < 0.001) and higher for probes matching the second stimulus (mean = 81.4%) as compared to the first stimulus (mean = 76.8%, t(22) = 6.85, p < 0.001, Fig. 2B).

To test whether working memory representations are attended in a theta-rhythmic fashion, we measured participants’ reaction times to matching probes at several densely sampled timepoints. We hypothesized that RTs should be modulated over time at a theta rhythm, indicative of rhythmic attentional fluctuations of the corresponding item in working memory. Furthermore, we hypothesized that RT time series for responses to valid and invalidly cued probes should be modulated in antiphase, suggesting that attention periodically alternates between the two items in working memory (Fig. 1B).

### Original analysis using traditional preprocessing and time-shuffling statistics

**Figure 3:**
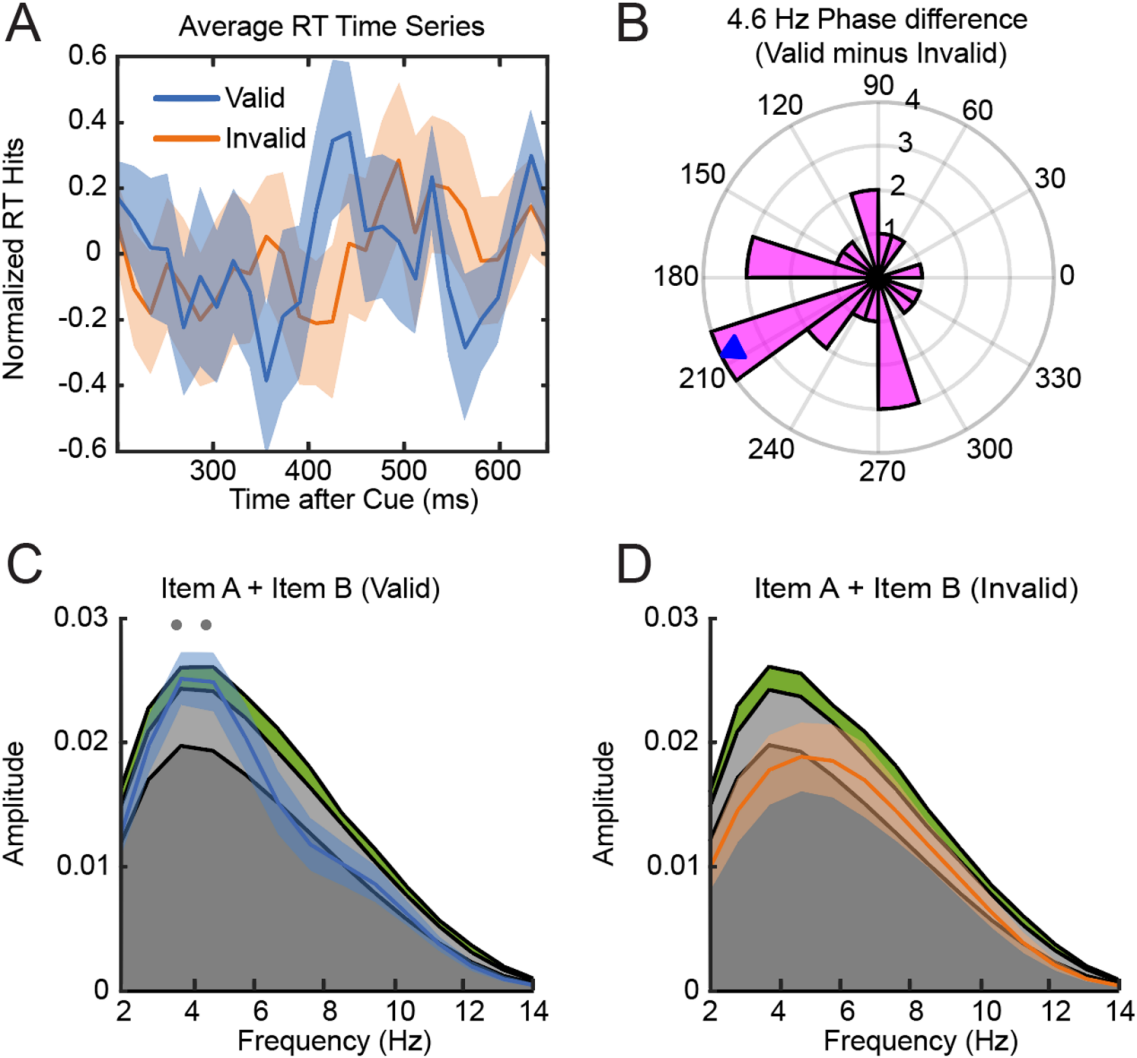
(**A**) Group average RT time series for valid and invalid trials. (**B**) 4.6 Hz Phase difference plot. (**C**) Power-spectrum RT time series valid condition. Colored areas in the background display quantiles of statistical null distribution (dark grey: Mean of permutation distribution, light grey: Power < 95% confidence interval, green: Power < 99% confidence interval). Dots indicate frequencies at which observed spectrum exceeded 95% of samples from permutation distribution (gray: significant before correction for multiple comparisons, black: significant after correction of multiple comparisons). (**D**) same as C but RT time series of Invalid condition.

To investigate rhythmic fluctuations in individuals’ responses, we calculated RT time series by averaging RTs in each of 30 SOAs for responses to validly and invalidly cued items respectively. The RT time series averaged over all subjects is shown in (Fig. 3A). Notably we did not perform the spectral analysis on the averaged time series in Fig. 3A but on individual RT time series and then averaged the resulting individual power spectra. This allowed us to ignore individual differences in phase and slight shifts in frequency that would have averaged out when averaging time series in the time domain. Comparing the averaged power spectra to the time-shuffled null distribution revealed a significant peak at 3.75 and 4.6 Hz in the valid condition and no significant peaks in the invalid condition (Fig. 3C,D). Because of differences in the number of collected trials for valid (75%) and invalid (25%) conditions we might simply lack the statistical power to detect rhythms in the invalid condition. In addition, a recent similar study on behavioral oscillations has also failed to find a significant peak in the power spectra of one of two conditions, revealing however that both conditions were still fluctuating in antiphase when analyzing individual oscillatory phases^32^. We therefore investigated whether our two conditions were in antiphase by analyzing phase difference between both conditions, as the difference angles should point towards 180 degrees if the oscillations are modulated in antiphase. The phase analysis of individual valid and invalid time series revealed that the mean of the 4.6 Hz difference angles was not significantly different from 180°, indicating an anti-phasic relationship of the oscillatory 4.6 Hz components (Fig. 3B). This result is indicative of attention periodically alternating between the two items over time.

**Figure 4:**
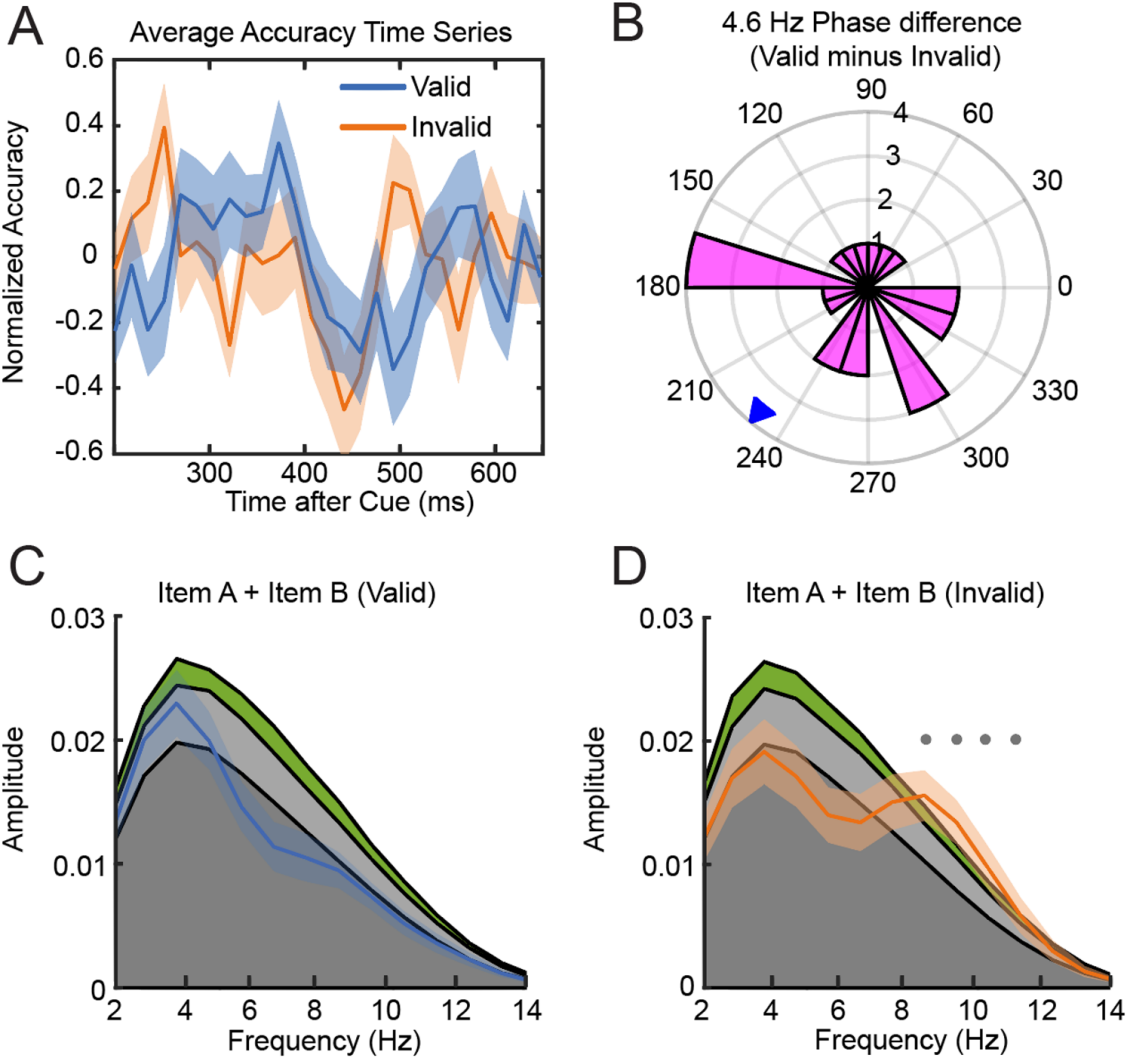
(**A**) Group average accuracy time series for valid and invalid trials. (**B**) 4.6 Hz Phase difference plot. (**C**) Power-spectrum accuracy time series valid condition. Colored areas (dark grey: mean of permutation distribution, light grey < 95% confidence interval, green < 99% confidence interval). (**D**) Same as (**C**) but accuracy time series of Invalid condition. Dots indicate frequencies at which observed spectrum exceeded 95% of samples from permutation distribution (gray: significant before correction for multiple comparisons, black: significant after correction of multiple comparisons).

We investigated rhythmic fluctuation in accuracy time series in an identical fashion by comparing observed average power spectra with stochastic null-distributions (Fig. 4A). The spectral analysis of individual accuracy time series revealed a significant peak at 8.4, 9.4 and 10.3 Hz in the invalid condition but no significant modulation in the valid condition (Fig. 4C,D). The phase difference between both conditions was significantly different from 180° indicating no anti-phasic relationship of 8.4, 9.4, 10.3 or 4.6 Hz components (Fig. 4B). We subsequently applied Rayleigh’s test of non-uniformity which revealed that phase differences were randomly distributed around the unit circle (p = 0.34) suggesting that fluctuations in accuracy for item A and B had no consistent temporal relationship.

#### Reanalysis using pipeline and AR(1) statistics suggested by Brookshire^35^

**Figure 5:**
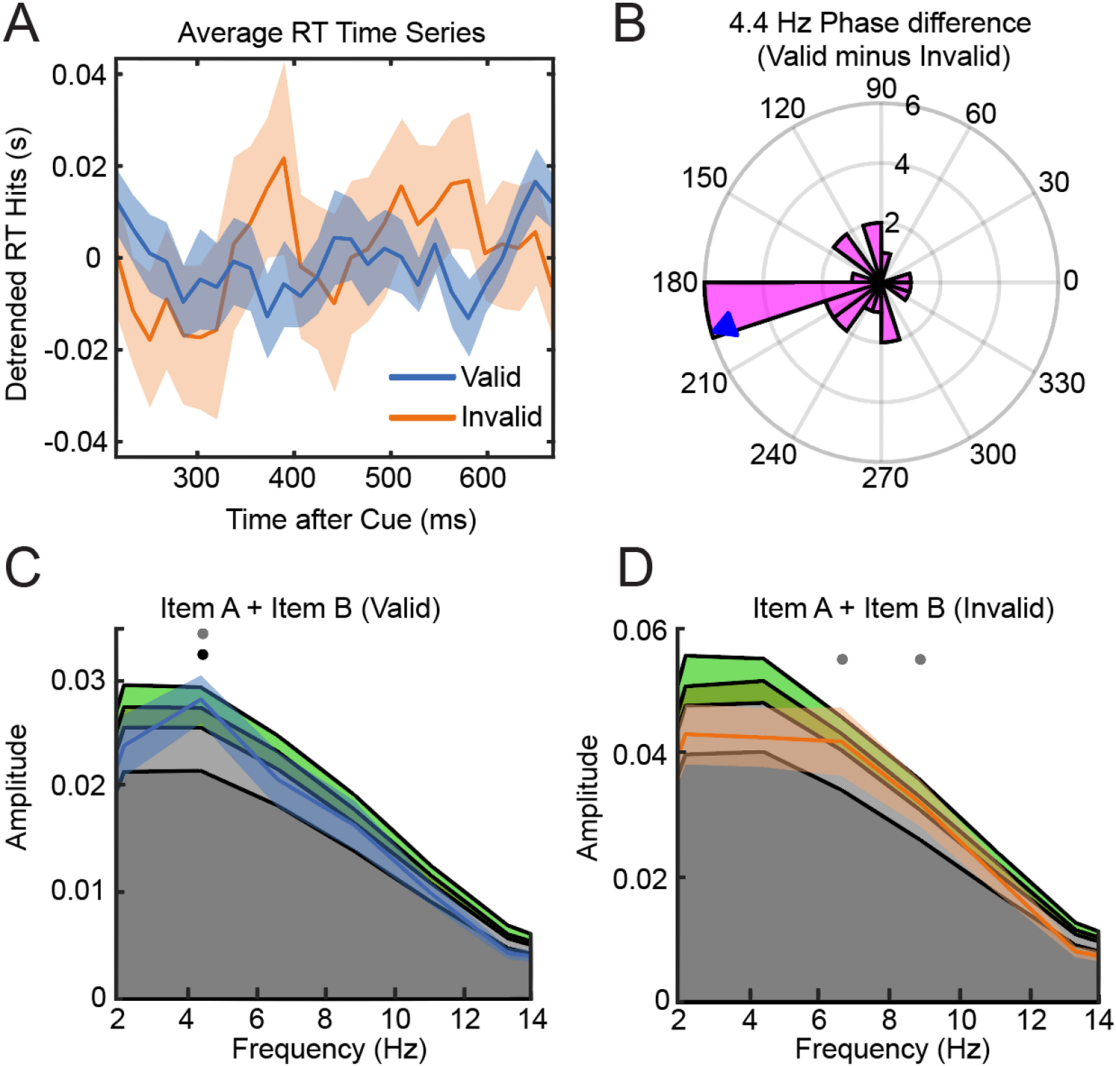
(**A**) Group average RT time series for valid and invalid trials. (**B**) 4.4 Hz Phase difference plot. (**C**) Power-spectrum RT time series valid condition. Colored areas in the background display quantiles of statistical null distribution (dark grey: Mean of permutation distribution, light grey: Power < 95% confidence interval, green: Power < 99% confidence interval). Dots indicate frequencies at which observed spectrum exceeded 95% of samples from permutation distribution (gray: significant before correction for multiple comparisons, black: significant after correction of multiple comparisons). (**D**) Same as C but RT time series of Invalid condition.

Performing spectral analysis on individual RT time series and averaging the resulting power spectra revealed a significant peak at 4.4 Hz in the valid condition (p = 0.03, fdr corrected) and no significant peaks in the invalid condition (Fig. 5C,D). The phase analysis of individual valid and invalid time series revealed that the mean of the 4.4 Hz difference angles was not significantly different from 180° (circular t-test: p < 0.001) and not uniformly distributed (Rayleigh’s test for non-uniformity, p = 0.017), indicating an anti-phasic relationship of the oscillatory 4.4 Hz components (Fig. 5B).

**Figure 6:**
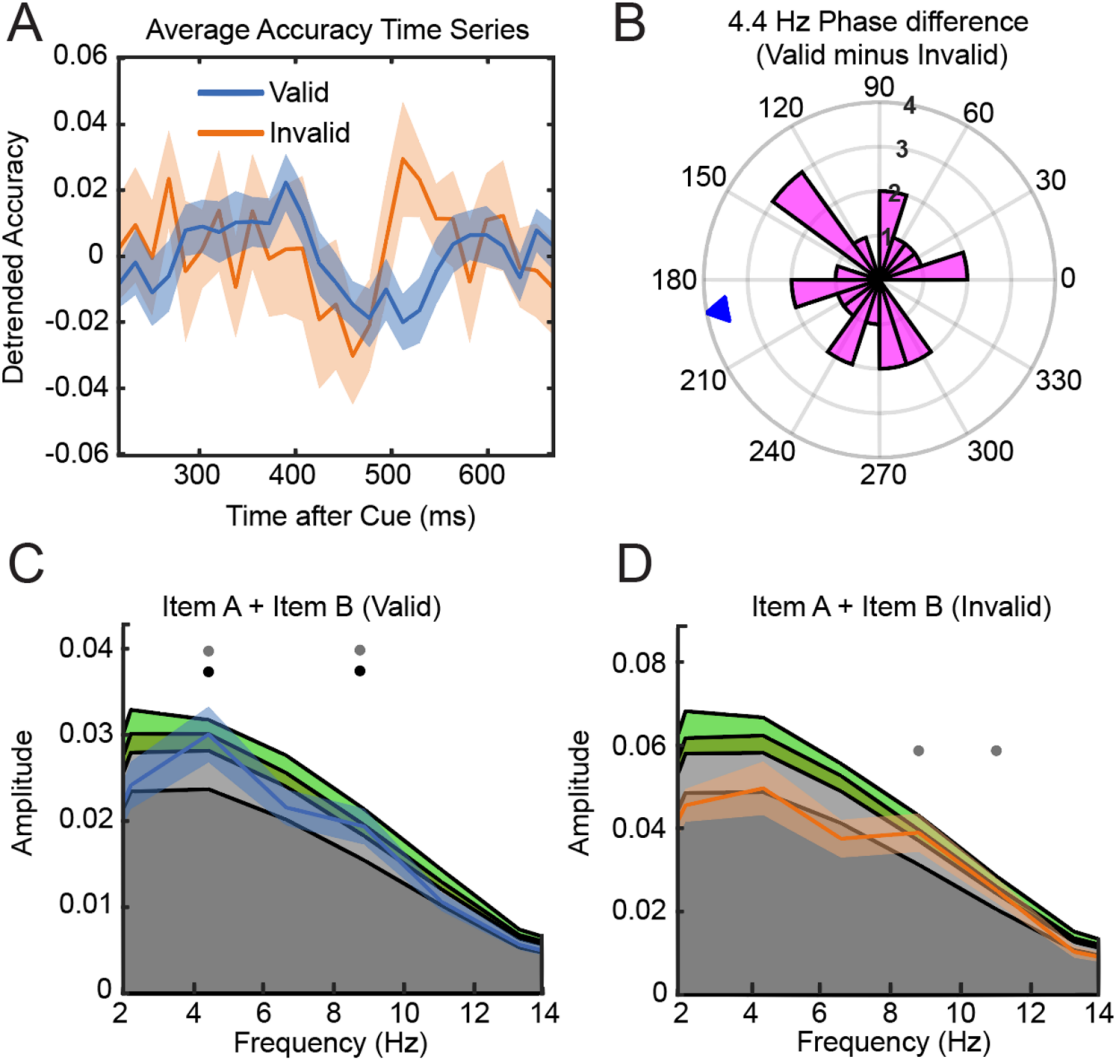
(**A**) Group average accuracy time series for valid and invalid trials. (**B**) 4.4 Hz Phase difference plot. (**C**) Power-spectrum accuracy time series valid condition. Colored areas (dark grey: mean of permutation distribution, light grey < 95% confidence interval, green < 99% confidence interval). (**D**) same as C but accuracy time series of Invalid condition. Dots indicate frequencies at which observed spectrum exceeded 95% of samples from permutation distribution (gray: significant before correction for multiple comparisons, black: significant after correction of multiple comparisons).

The spectral analysis of individual accuracy time series revealed significant peaks at 4.4 Hz and 8.9 Hz in the valid condition but no significant periodicities in the invalid condition (Fig. 6C,D). Valid-invalid phase differences of 4.4 Hz components were uniformly distributed (Rayleigh’s test for non-uniformity, p = 0.824, (Fig. 6B) indicating no anti-phasic relationship.

### Comparison of outcomes

Applying two distinct techniques to analyze behavioral time series allowed us to validate our results and compare similarities and differences between both methods. As the main purpose of this paper was to investigate rhythmic fluctuations in behavior, we will not conduct a systematic in-depth comparison of both techniques but only provide a qualitatively comparison of the main results related to our initial hypothesis.

Both analysis techniques confirmed our main hypothesis consisting of 1. A rhythmic modulation of behavioral outcomes in the 3 to 6 Hz range and 2. An anti-phasic relationship between valid and invalid behavioral time series. Importantly however the results also differed in several aspects. Whereas both time shuffling (TS) and autocorrelation models (AR(1)) revealed significant rhythms in valid RT time series at 4.6 and 4.4 Hz respectively, this effect was only significant before correction for multiple comparisons in the TS analysis. Furthermore, AR(1) showed a significant peak at 4.4 Hz in valid accuracy time series that was not uncovered by TS.

Overall, the AR(1) technique led to results that were more pronounced and more in line with our predictions. We will discuss the implications of our comparison further in the discussion.

## Discussion

Object-based as well as feature-based attention to visually available information have been demonstrated to undergo rhythmic fluctuations in the theta (3 to 8 Hz) range. Here we set out to test whether attention directed towards multi-feature items in working memory is modulated in a similar theta rhythmic manner. Using the analysis pipeline proposed by Brookshire^35^, we find that (a) the speed and accuracy of responses to cued probes that match one of two items in working memory is modulated at a frequency of ~ 4.4 Hz and (b) rhythmic fluctuations of reaction times to cued and un-cued items are modulated in antiphase. Our results are indicative of a rhythmic and alternating attentional sampling of both multi-feature working memory representations, which in turn modulates response speed and accuracy of subsequent probe comparison. We would like to note that in the remainder of the manuscript we will only discuss the findings that resulted from the analysis approach proposed by Brookshire^35^ using minimal preprocessing and the AR(1) technique. While both methods had partially different outcomes, our main hypothesis (theta fluctuations in behavioral time series and phase opposition between valid and invalid time series) was confirmed by both.

### Rhythmic fluctuations in behavior

While rhythmic fluctuations in RT and accuracy time series were observed for valid trials, we did not find a significant oscillatory peak in trials in which participants were invalidly cued after correction for multiple comparisons. However, when analyzing the individual 4.4 Hz phase of valid and invalid RT time series we found a significant anti-phasic relationship. This suggests that an oscillatory component was also present in invalid trials which the conventional time-shuffling statistical analysis failed to reveal. We propose several possible reasons for this. First, since cue validity was 75% percent, invalid time series were calculated from fewer trials than valid time series which might have led to increased noise and a worse estimation of oscillatory components (average number of trials in RT time series per participant: valid = 350.3, invalid = 100.6). Additionally, it is possible that the cue (75% validity) led to an attentional de-prioritization of the un-cued item. We will discuss this particular possibility in more detail in the section below (see ‘Comparison to other studies’). It is also noteworthy that we did not observe significant periodicities when performing spectral analysis directly on the grand average time series (Figs. 3A, 4A, 5A, 6A). We speculate that this might be caused by slight differences in the phase of individual oscillatory time series that cancel out when averaging the data of all subjects. Importantly however, valid and invalid time series still fluctuated in antiphase on an individual level which could be explained by individual differences in the speed of attentional shifts following the retro cue. On average retro-cue benefits tend to fully manifest after 300 to 500 ms, however little is known about how much this varies between participants^19^. Future experiments should investigate individual differences in the speed of attentional allocation since this might have important implications for the study of internal attentional sampling.

The phase analysis (4.4 Hz) of RT time series revealed that behavior fluctuated in phase opposition between valid and invalid conditions. This pattern is indicative of attention initially being allocated to the cued item and then sampling both items in working memory alternatingly at a theta frequency. Importantly this anti-phasic dynamic is unlikely to be coincidentally caused as a result of the timing or order with which the stimuli were presented, e.g., by the hypothetical initiation of two independent rhythmic processes at the onset of the first and second stimulus. If this was indeed the case, as we calculated valid and invalid RT time series each from equal amounts of trials in which the first and second stimulus was probed, coincidental anti-phasic periodicities should average out. In contrast we find consistent oscillatory power for valid and an anti-phasic relationship between valid and invalid trials, speaking clearly against this potential confound.

In addition to the 4.4 Hz fluctuation, our analysis of accuracy time series revealed a significant rhythmic modulation at 8.9 Hz in the valid condition. Rhythmic fluctuations of behavioral time series at 10 Hz are frequently observed in behavioral studies (see VanRullen^1^, for a review). As this study was mostly interested in behavioral rhythms in the theta range, we will not thoroughly discuss these effects. We would like to note however that Pomper and Ansorge^32^ also found accuracy time series to be modulated at 9.7 Hz, indicating that this ~ 10 Hz alpha rhythmicity might be an additional signature of periodic processing of perceived or memorized visual representations.

### Comparison to other studies

How do our results relate to other studies investigating rhythmic attentional fluctuations in working memory^31,32^?

The two previous studies have found behavior to oscillate at 6 Hz, while we find behavioral oscillations at 4.4 Hz. One hypothetical explanation for this discrepancy might be the difference in stimulus complexity between studies. Multi-features objects in working memory, as the ones used here, might require a more effortful and longer activation process leading to less frequent sampling per item.

Whereas our study and the study by Peters et al.^31^ used an explicit visual cue to reset attentional rhythms, Pomper and Ansorge^32^ induced attentional sampling via the presentation of the second object. In both cueing studies the cue had a validity of 75% giving participants a clear incentive to prioritize one item over the other, whereas both items should be equally prioritized in the study by Pomper and Ansorge^32^. Importantly however, Pomper and Ansorge^32^ found faster reaction times and higher accuracy for the second item, in line with a recency effect which has been hypothesized to be caused by attentional prioritization^38^. Interestingly, both our and Pomper & Ansorge’s studies observed significant rhythmic fluctuations relative to the reset event only for the prioritized item. Although in our case this might be explained by lower trial numbers, the same pattern has been reported in another study on external attentional sampling using much larger number of trials^2^. One potential explanation for this might be that attention exerts stronger rhythmic effects on the prioritized item, causing high amplitude modulations in behavioral time series, whereas the concurrent rhythmic sampling of non-prioritized items has weaker periodic effects on behavior. Alternatively, it has been proposed that attention might be governed by a central sampling mechanism or “master sampler” between 7 and 10 Hz that can be split between two objects^3,39^. A hypothetical 10 Hz sampler could thus sample both items 5 times per second but could also sample the prioritized item more frequently (e.g. prioritized sampled 8 times and nonprioritized is sampled 2 times per second). One could speculate that in spectral analysis of valid time series this would be evident in periodicities at the fundamental frequency (10 Hz) as well as its harmonics (5 Hz) which are indeed observed in our data as well as the data of Pomper and Ansorge^32^. Importantly both of these possibilities, stronger or more frequent sampling, assume that attention actively prioritizes either one or the other item.

A slightly different interpretation, relating to work by Fiebelkorn et al.^2^ was proposed by Peters et al.^31^ who found rhythmic fluctuations between objects in working memory. Similar to the findings by Fiebelkorn et al.^2^ however, reaction times to targets presented at different-object locations were periodically equal, but never faster than the ones presented at same-object locations, indicating that attention never completely switched to the other object. This suggests that object-based attention rhythmically alternates between phases in which the entire cued object is prioritized (and the un-cued object de-prioritized) and phases where cued and un-cued objects are prioritized equally. According to this view attention would not prioritize both objects in alternation but would remain focused on a single object. Our results support this view insofar that reaction times in invalid trials were almost never as fast as those in valid trials, suggesting that attention never really seemed to switch completely to the other item in WM. However, it has to be noted that this argument relies on the assumption that the rhythmic attentional effects are the only factors influencing reaction times. Attentional cues might change the status of items in working memory in a more continuous fashion, e.g., by increasing baseline activation, in addition to which we could be observing effects of rhythmic attention. Future studies manipulating cue validities in a more systematic way will have to shine light on this open question.

### Functional significance and neural mechanisms

There are several functional processes that could explain the rhythms found in our study. Periodic attentional refresh of working memory representations could be a fundamental requirement for WM maintenance. Evidence from computational modeling studies have proposed that such periodic reactivation, or “replay”, might underly working memory maintenance and could be implemented by an interplay of theta (4 to 8 Hz) and gamma (30 to 80 Hz) oscillations^40–42^. Periodic attentional refresh has also been proposed to counteract time-based decay in visual working memory. Evidence for time-based decay however has not been conclusive^43,44^.

It has also been proposed that rhythmic attentional prioritization might help to solve the binding problem by co-activating stimulus features into coherent representations^45^. An important aspect of our study that distinguishes it from previous work is the fact that we used stimuli that consisted of multiple relevant features (colour + orientation) which had to be encoded together. Because we presented both items at fixation we could investigate effects of non-spatial object-based attention and found that similar rhythms occur for non-spatial features. The effects of the attentional cue on average accuracy indicate that colour-based retrieval also led to a prioritization of the orientation feature bound to that item, as orientation was the feature that had to be compared with relatively high precision (as compared to the easily distinguishable colours). More importantly we also show that colour cues led to rhythmic fluctuations in accuracy measures. Together with the fact that probes could only be compared based on both colour and orientation we hypothesize that attention cyclically prioritized both features of an item. Moreover, since both items fluctuated in antiphase, they were both rhythmically prioritized but never concurrently. In working memory this would allow the visual system to keep color features bound to the corresponding orientation features by activating the correct combination of features one after the other, without risking co-activation of two unrelated features. This process, commonly referred to as time-based multiplexing has been proposed to prevent merging of separate neural representation and might aid WM readout^42,46–49^.

In the past years, theories have tightly linked external attentional selection with neural oscillations^8,9,50,51^. Even more recently the fundamental relationship between internal attention towards working memory representations and oscillatory activity has become evident^52–55^. Current models propose that internal attention is implemented by a dynamic interplay between frontal theta oscillations, acting as a top-down control mechanism and occipital alpha oscillations that inhibit or disinhibit single representation. Our findings might be explained by a similar mechanism that modulates relative activity between two more or less equally relevant items on a shorter timescale.

### A common rhythmic sampling mechanism for external and internal visual representations

Last, we would like to propose a hypothesis that could potentially explain some of the commonalities between external and internal sampling rhythms found in most recent work. We believe that a general attentional sampling mechanism operating at a frequency of ~ 10 representations per second might underly the behavioral fluctuations found in internal and external attention tasks. Several studies have already provided convincing evidence that attention to external stimuli is fluctuating in the theta range^2–5^. Most studies converge on a frequency of ~ 4 to 6 Hz with which 2 items are sampled. Importantly it was also demonstrated that extending the number of attended locations to 3 reduces the frequency at which individual items are sampled to ~ 3 Hz^3^. It was already hypothesized that this observation could support the existence of a single external attentional sampler in the ~ 10 Hz range that has the capacity to highlight 10 perceived items/locations per second^3,39^. Our study, as well as the work from Pomper and Ansorge^32^ and Peters et al.^31^, has demonstrated that this rhythmic attentional sampling process extends to internal information that is maintained in working memory. Importantly, much evidence for the sensory recruitment hypothesis has been provided, suggesting a shared substrate between perception and working memory^23,26,27^. External and internal attention would therefore have to access representations that are encoded in highly similar areas with strongly overlapping codes. It is hence conceivable that a common attentional “master” sampler in the 10 Hz range rhythmically boosts activity in specific neural representations irrespective of whether they reflect information that is currently perceived or encoded in working memory. Further investigations will have to show if this model of attention holds true.

### Comparing time-shuffling and AR(1) analysis pipelines

The recent publication by Brookshire^35^ critically addressed the preprocessing pipeline and statistical time-shuffling procedures used by the vast majority of publications in the behavioral oscillations field by demonstrating the poor control over type-1 errors. We analyzed our data with both, original time-shuffling and newly proposed AR(1) technique and show that both methods revealed significant rhythmic fluctuations in behavior. While some oscillatory peaks found by time-shuffling did not reach significance when using the AR(1) method, other peaks that we had predicted based on previous literature were statistically more pronounced or only reached significance when using the AR(1) technique.

While our aim was not to systematically compare time-shuffling and AR(1) methods, our analysis comparison still provides relevant insights into the current debate on the right statistical procedure^35^. First, our main results consisting of fluctuations in the 4–5 Hz range and phase opposition, persisted between both analysis pipelines, showing that time-shuffling does reveal veridical effects. Although the four re-analyses attempted by Brookshire^35^ did not reveal significant results, it has to be noted that the AR(1) method can be significantly less sensitive (as compared to time-shuffling) to genuine effects, depending on task parameters like signal length and sampling rate. Although AR(1) might be a statistically more valid test, many findings in the rhythmic attention literature likely still reflect true results. Second, we found that using fewer preprocessing steps in combination with the AR(1) technique revealed results that were more in line with our predictions. Although we can obviously not say with absolute certainty that our effects are genuine, the AR(1) method might allow to decrease the number of preprocessing steps while still providing sufficient sensitivity, and hence provide a more unbiased assessment of rhythmicity in the data.

We suggest caution in disregarding previous work too quickly and recommend future projects to report results based on both analysis techniques.

### Conclusion

In conclusion, we add to the evidence that attention samples multi-feature objects in working memory at a theta rhythm. This rhythmic attentional facilitation fluctuates between two relevant items in working memory, evident in anti-phasic modulation of behavioral time series. We hypothesize that the brain might utilize this oscillatory time-multiplexing in the face of limited attentional resources, the binding problem and/or attentional refresh required for memory maintenance. The patterns found in this study closely mimic recently found rhythms in external spatial and feature-based attention that were shown to be tightly linked to frontal theta and occipital alpha oscillation. Identical oscillatory mechanisms have been linked to attentional control in working memory. We hypothesize a single, theta-rhythmic, attentional sampling mechanism for perceived and memorized representations.

## Supplementary Information

Supplementary Figure 1.

## Data Availability

All data conducted in the context of this study is freely available on osf.io/39g4w.
