# Supplementary material for: Attention rhythmically samples multi-feature objects in working memory
Source: Sci Rep. 2022 Aug 29;12:14703. doi: 10.1038/s41598-022-18819-z (PMC9424255; doi:10.1038/s41598-022-18819-z)
Supplement: Supplementary file 1 — Supplementary Figure 1. [file 41598_2022_18819_MOESM1_ESM.docx]

Supplementary Materials


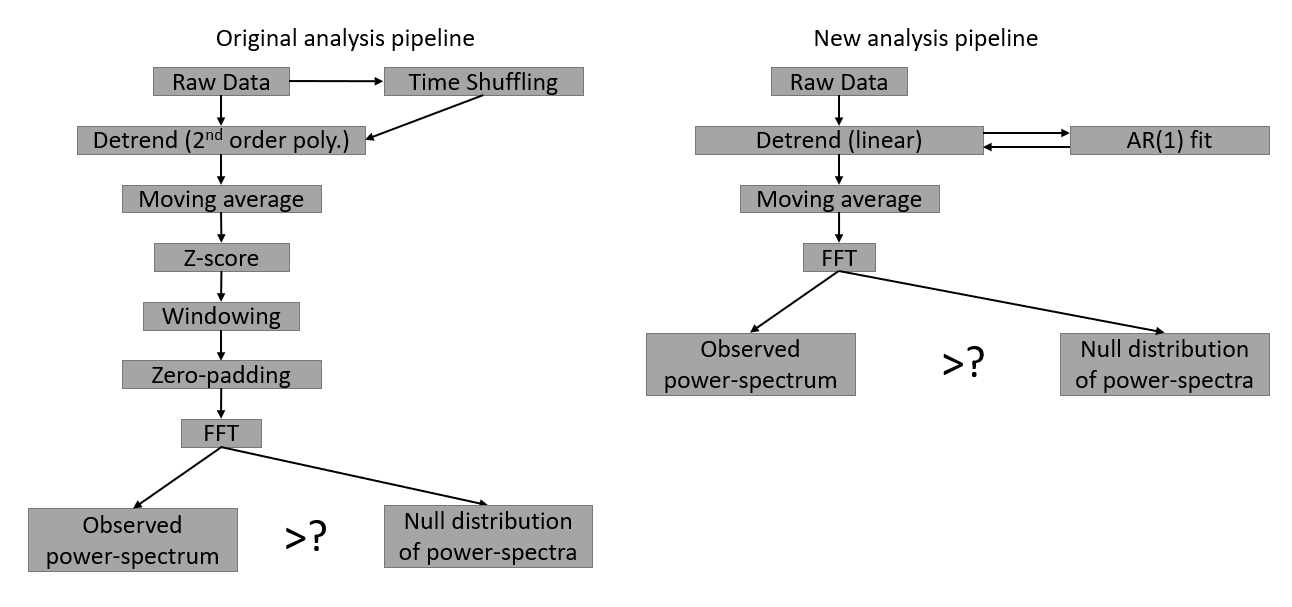


[Supplementary Figure 1. Visual comparison of the traditional analysis pipeline (left) and the procedure suggested by Brookshire (2022) (right)]
